# An exploration of migrant women’s perceptions of public health messages to reduce stillbirth in the UK: a qualitative study

**DOI:** 10.1186/s12884-021-03879-2

**Published:** 2021-05-20

**Authors:** Tomasina Stacey, Melanie Haith-Cooper, Nisa Almas, Charlotte Kenyon

**Affiliations:** 1 School of Human and Health Sciences, University of Huddersfield, Huddersfield, United Kingdom; 2 Calderdale and Huddersfield NHS Foundation Trust, Lindley, Huddersfield United Kingdom; 3 Faculty of Heath Studies, University of Bradford, Bradford, United Kingdom

**Keywords:** Pregnancy, public health, stillbirth prevention messages, Black, Asian and Minority Ethnic women, migrant

## Abstract

**Background:**

Stillbirth is a global public health priority. Within the United Kingdom, perinatal mortality disproportionately impacts Black, Asian and minority ethnic women, and in particular migrant women. Although the explanation for this remains unclear, it is thought to be multidimensional. Improving perinatal mortality is reliant upon raising awareness of stillbirth and its associated risk factors, as well as improving maternity services. The aim of this study was to explore migrant women’s awareness of health messages to reduce stillbirth risk, and how key public health messages can be made more accessible.

**Method:**

Two semi-structured focus groups and 13 one to one interviews were completed with a purposive sample of 30 migrant women from 18 countries and across 4 NHS Trusts.

**Results:**

Participants provided an account of their general awareness of stillbirth and recollection of the advice they had been given to reduce the risk of stillbirth both before and during pregnancy. They also suggested approaches to how key messages might be more effectively communicated to migrant women.

**Conclusions:**

Our study highlights the complexity of discussing stillbirth during pregnancy. The women in this study were found to receive a wide range of advice from family and friends as well as health professionals about how to keep their baby safe in pregnancy, they recommended the development of a range of resources to provide clear and consistent messages. Health professionals, in particular midwives who have developed a trusting relationship with the women will be key to ensuring that public health messages relating to stillbirth reduction are accessible to culturally and linguistically diverse communities.

**Supplementary Information:**

The online version contains supplementary material available at 10.1186/s12884-021-03879-2.

## Background

Perinatal mortality is a global public health issue and there are wide variations in the rates of stillbirth across the world, even between and within high income countries [1]. For the last 5 years reducing stillbirth has been a national priority within the United Kingdom [UK] and there has been a 16 % reduction from 4.2 stillbirths per 1000 total births in 2013 to 3.5 per 1000 in 2018 [2]. However there remains a considerable disparity in outcome within the population, with Black, Asian and Minority Ethic [BAME] and in particular migrant women continuing to have higher rates of stillbirth compared to white women [2]. The reasons for these inequalities in stillbirth rates are not clear, but are likely to be multifaceted and include underlying structural racism, socio-economic disadvantage and associated stress and coping mechanisms, access and acceptability of health care and accessibility to public health messages around reducing perinatal mortality [3, 4] Previous research has identified that migrant women’s experience of maternity care in the UK is generally poorer than that of women born in the UK [5, 6].

Raising awareness of public health messages is an integral part of maternity care and an important element in improving perinatal outcome [7]. A number of potentially modifiable risk factors for stillbirth have been identified in the last few years, including: optimal body mass index prior to conception [8], smoking cessation in pregnancy [9], screening for gestational diabetes [10], awareness of reduced fetal movements [11], maternal side going-to-sleep position in pregnancy [12], and reducing caffeine intake in pregnancy [13]. There has been a concentrated focus on reducing stillbirths in the UK in the last ten years and NHS England’s ‘Saving Babies Lives Care bundle’ forms a part of this strategy, within the second version there are key ‘safe and healthy ‘ pregnancy messages aimed to support conversations with women in pregnancy reading a range of [14]. However, it is unclear whether key health messages relating to these factors are available, accessible and appropriate for diverse communities within the UK, especially women who may experience language and cultural barriers when accessing maternity care. This study aimed to examine migrant women’s awareness of stillbirth and health messages relating to reduction of stillbirth risk in the UK and to explore women’s views on how to develop culturally and linguistically appropriate interventions to deliver the key messages around stillbirth prevention.

## Methods

 Ethical approval was gained from the University ethics committee SREIC/2019/132 and a qualitative study was undertaken between November 2019 and May 2020. After acquiring informed consent, women were recruited to the study through “gatekeepers” at local voluntary sector organisations. Women were eligible to participate if they were from a BAME community, had migrated to the UK at some point in their life, were aged over 18, had a child under the age of five born in the UK and spoke either English or a language where a voluntary sector interpreter could be arranged. This study aimed to examine migrant women’s awareness of public health messages relating to reduction of stillbirth risk in the UK and to explore culturally and linguistically appropriate interventions to deliver the key messages around stillbirth prevention. The focus was on migrant women as they may be less integrated into UK life, for example not having experienced the UK education system, so may face more barriers to accessing health service. Women who had previously experienced an adverse outcome were excluded from the study. The gatekeepers were provided with study information, interested women were then invited by the research team to attend a face to face focus group or, after restrictions due to COVID-19, to undertake individual telephone interviews.

Purposive sampling was undertaken to ensure the sample included women from various countries of origin, different cultural backgrounds and with different levels of English language literacy. Before the COVID-19 pandemic, two focus groups of women were conducted. These were undertaken in English in community centres, with established community groups. The purpose was for 8–12 women to explore the questions in depth and for the group dynamics to facilitate mutual support, encourage quiet participants to contribute and to lead to further depth of exploration of the issues under discussion [15]. During the COVID-19 pandemic, interviews were arranged on a one to one basis over the telephone. The same semi-structured interview guide was used for the focus groups and interviews. The questions related to knowledge of stillbirth, awareness of health messages around reducing stillbirth risk in pregnancy and who communicated these messages. Further questions explored what participants believed would be the best way to communicate such messages in the future. Probes were used to explore the issues in more detail. See Supplementary Table 1 for the interview guide.

## Analysis

Interviews were audio recorded, transcribed verbatim and thematic analysis was conducted, using Word and the highlighting functions, following the principles from Braun and Clarke [16]. Two members of the team [MC and TS] read all the transcripts, searching for patterns in the data to develop codes which were then used to construct themes. Other members of the team also read the transcripts and checked the analysis process. Discussion took place until consensus was reached on the final themes.

## Results

In total, 30 women took part in the study, seventeen [in groups of 9 and 8] took part in two face to face focus groups and a further thirteen took part in individual interviews via telephone due to the COVID-19 pandemic. They had all given birth in one of four North of England Trusts [see Table 1 for full details].

**Table 1:** Participant profile

Participant	Type of interview	Home country	Time in UK	Age	Parity
1. FG 1	Focus group	Pakistan	8 years	34	P2
2. FG1	Focus group	Pakistan	2 years	30	P1
3. FG1	Focus group	Pakistan	5 years	23	P2
4. FG1	Focus group	Pakistan	3.5 years	24	P1
5. FG1	Focus group	Bangladesh	1 year	26	P1
6. FG1	Focus group	Pakistan	1 year	27	P1
7. FG1	Focus group	Hong Kong	2.5 years	24	P1
8. FG1	Focus group	Pakistan	1.5 years	27	P2
9. FG1	Focus group	Pakistan	2 years	25	P5
10. FG2	Focus group	Ethiopia	7 years	32	P4
11. FG2	Focus group	Not stated	3 years	39	P4
12. FG2	Focus group	Guinea	1 year	30	P2
13. FG2	Focus group	Somalia	5 years	40	P7
14. FG2	Focus group	Tunisia	10 years	32	P2
15. FG2	Focus group	Congo	3 years	26	P3
16. FG2	Focus group	Congo	2 years	30	P2
17. FG2	Focus group	Congo	2.5 years	30	P2
18.	Individual telephone [with interpreter]	Saudi Arabia	6 years	36	P1
19.	Individual telephone	Egypt	2.5 years	38	P4
20.	Individual telephone	Yemen	2 years	32	P1
21.	Individual telephone	Pakistan	4 years	32	P1
22.	Individual telephone	Russia	19 years	30	P3
23.	Individual telephone	Albania	1.5 years	24	P1
24.	Individual telephone	Sudan	3 years	29	P1
25.	Individual telephone	Ethiopia	11 years	31	P1
26.	Individual telephone	Iran	6 years	36	P2
27.	Individual telephone	Uganda	5 years	22	P1
28.	Individual telephone	Senegal	9 years	33	P1
29.	Individual telephone	Sudan	10 years	31	P3
30.	Individual telephone	Eritrea	3 years	28	P1

Four main themes emerged from the data: awareness of stillbirth, awareness of health messages to keep baby safe in pregnancy, the role of health professionals and making health messages accessible

1. Awareness of stillbirth

Women were asked what they understood by the word ‘stillbirth’. Most women had never heard the word in English and for some the word ‘stillbirth’ did not exist, or they had not come across it, in their first language:

*‘Naturally die, no I never heard about it, no, I’m not sure if we have any word in my language about it, no.’* P26 Iran.

Some women discussed how stillbirth may be described in their first language:

*‘when the baby born and he’s dead, they just say the baby’s dead, they don’t have like a word like the stillbirth’* P22 Russia.

In addition, some women discussed how, culturally, there was a silence around stillbirth, a lack of discussion or sharing of information about why it might happen:

*‘When the neighbours used to come around and I would hear that there’s been a death but we never found out what happened, how it happened just they used to say Oh baby died in the stomach.’* P21 Pakistan.

*‘So it’s not something that’s discussed. … but I’ve seen people talk about on social media about their experiences and post something about angel day where they celebrate the kids who were never born or who were born obviously as a stillbirth. So I think it’s all cultural. I’ve never heard of anything like that in Russia but it’s definitely a little bit more right here in England.’* P22, Russia.

Many women discussed how the topic of stillbirth can be frightening and how discussing or referring to stillbirth in pregnancy could increase stress levels in women who may already be living difficult lives.

***‘****I think it’s best that you do not mention stillbirth…to a woman who is pregnant [antenatal] review. I have seen both sides ladies are pregnant and have problems at home thinking all the time …this might happen to me or it might not happen to me.* P28, Senegal.

In addition, some women discussed the idea of fate and that stillbirth will just happen with no underlying reason, therefore mentioning it would cause stress in women unnecessarily:

‘I mean if it happened it happened…you won’t need to hear about it…just thinking all your pregnancy…scared and stressed…… most of the ladies they say there is no reason for that.’ P24, Sudan

2. Awareness of health messages to keep baby safe in pregnancy

Although participants we often not aware of the term ‘stillbirth’ and did not remember it being discussed in pregnancy, when questioned about specific advice they may have received about keeping their babies safe, they were frequently knowledgeable about key messages such as being aware of fetal movements and going to sleep on their side:

*‘I remember the midwife used to inform me any time about how to track the baby’s movement, to see if the baby’s moving okay. She suggested not to take any medication without doctor’s prescription, so to protect the baby. And also not to drink alcohol, not to take drugs, not to smoke.’* P23 Albania.

*‘Yeah, they said not sleep on your back.’* P24 Sudan.

However they did not generally associate these messages with stillbirth reduction:

*‘I don’t think I remember anything about while I was pregnant to prevent child stillbirth… the ninth month and I was told maybe sleep onto your side just because you can breathe better …but it wasn’t about the child.’* P20, Yemen.

Many of the women discussed how they received advice about keeping themselves and their unborn baby healthy. Advice received from family or friends was often different to the advice received by health professionals in the UK. This included dietary advice:

‘*my family said you are now two, not one person, so you have to eat a lot, as baby needs it*…*and during the first three months they said I don’t have to eat some things like banana…the food that is warm, especially they mention banana or date and then after three months yes again because it’s boy I have to be careful about my diet, I’m not allowed to eat fish and just salmon, and seabass…’* P26, Iran.

Women were offered advice from family and friends as well as health professionals about all aspects of daily life in order to keep themselves and their unborn baby healthy, there was a wide variation in the messages that women received:

*‘when I go [*to church*], they said sit here, don’t stand up a lot, not too much, too long time, you have to sit here, they told me like that, rest, like don’t stand in our church…’* P30 Eritrea.

***‘…****they told me you have to work to work, work, work because it’s good for you, don’t stay at home…’* P30 Eritrea.

The different advice was not always consistent and this was sometimes confusing:

*So I think the midwife would say “you’re only allowed so much tuna a week” and then people in Russia would be like “oh I don’t know what you’re talking about, you can eat as much of this as you want…So it’s just little things like that that would get confusing’* P22, Russia.

It was also noted, that some of the advice that women appeared to receive from health professionals, was not always in line with current national guidance: This particularly related to the urgency of seeking support if there was a perception of decreased fetal movements:

*‘Yes, she asked me if I don’t feel any movement for two days I think, if I remember, two days I have to go to the hospital’* P26, Iran.

*‘And then sometimes I’m calling for midwife and then they told me eat like ice cream, like ice, you need to cold the baby and then they move.*’ P25, Ethiopia.

3. The role of health professionals

All the women discussed how they received advice from their midwife in pregnancy around staying healthy. Women believed that targeted information to reduce their risk of stillbirth should be primarily communicated through health professionals, in particular midwives. Midwives, nurses, doctors, GPs and health visitors were all named as key people with this responsibility. Women believed that the information from health professionals was the most trustworthy when compared to other sources:

*‘…the midwives maybe should advise more the clients, the patients, because at least in my culture, in my country, when a doctor says something or when a nurse says something, when a midwife, it’s more trustful. And the patient take it more seriously than they will take in information on internet or in a leaflet…’* P23, Albania.

Some women discussed how cultural and structural barriers reduced the likelihood of migrant women attending for routine appointments and this needed to be addressed as a key element of stillbirth reduction strategies:

*‘I think first of all we should advise them to go see a hospital…African communities, most of them, they don’t go to hospital because some of them, they give birth at home, by themselves. Because there are some countries people have to beg for hospital. And here, we have the chance that we can go to hospital, have a good midwife that can explain. Maybe the advice you should give them is first to trust to go to hospital first, to get checked.’* P28, Senegal.

Trust was considered an important issue for most women, especially those who did not have family and friends in the UK and women believed it was the responsibility of the midwife to build up a relationship to ensure that she trusted the advice given:

*‘I like to be explained that I know that you came from other country ….I have a really successful pregnancy with Daniel, with my son, because I just talk between, you know, two sides [midwife]’* P26, Iran.

4. Making health messages accessible

Participants suggested that it was important that women were aware about reducing the risk of stillbirth pre-conceptually as well as repeated throughout pregnancy to avoid women turning to less reliable sources of information:

*‘The best way is update the information…at least once a month because sometimes new mothers, they don’t know anything, they take any advice from anyone, maybe that advice is not suitable for them, you have to go to midwife, doctor is better than internet.’* P29, Sudan.

*‘Well, I think maybe it should be before. Sometimes some ladies, before they get pregnant they have use with drugs and alcohol, and that’s why it can make their baby die. So maybe it’s better to start before’* P23, Albania.

Women discussed how they had accessed information about pregnancy related issues from sources outside of maternity services and that this approach could be useful to complement, but not replace, the key messages around reducing the risk of stillbirth provided by health professionals. Many women accessed the internet, in particular the NHS website to source information related to pregnancy. However, this was thought to be too general for some of the information women required.

*‘But if for example it was a specific website for the women, I couldn’t find it, because NHS is for general information about everything you can find, but for example it was a website, specific for pregnancy, or post pregnancy, you know, it was really good, and we can trust*.’ P26 Iran.

Some women discussed how they relied on internet sources to corroborate the message provided by the health professional:

*‘they always check internet, while the midwife told them, or the family told them, but they always check the website’* P26 Iran.

*‘because my midwife was from Pakistan, and okay so from another culture, another experience and so I check the internet…’* FG2.

Some women discussed how written information would be useful to communicate key messages about reducing stillbirth. However, other women did not think written information was very helpful. Many did not, or could not, read the information provided by health professionals and would not read written key messages around reducing stillbirth. In addition, one woman talked about how some migrant pregnant women may have a stressful life which is not conducive to receiving written information:

*‘…most people I know, I think because you’re going through a lot of emotions and a lot of like things going on in your body, so people are weak, people are tired every time. I don’t think leaflets would be a good idea to just sit down and read.’* P27, Uganda.

Some women discussed how they had used mobile applications in pregnancy to access general advice. They suggested the idea of the key messages about stillbirth prevention being provided on a mobile application downloaded onto a woman’s smartphone. When asked about other ways of communicating key messages around stillbirth prevention some suggested that a video might be effective:

*‘films with graphics and things, that you can see not only hear, it might be a good way to spread that information, just because if you don’t understand what’s been saying you can have the photos or pictures and you can understand it’.* P21, Pakistan.

However, two women identified that they did not have regular access to the internet through a smartphone during their pregnancies, and it was felt that some women would need instruction on how to use an app.

Some women suggested that mobile phones could be used to provide text messages as a reminder about the key messages. However, other women thought that this may not be good for some women, as shown by this participant when asked specifically about the use of text messages:

*‘…some women maybe they will not care about the message you know, because it’s a message sometimes they delete it…’ P29, Sudan*.

Social media was suggested by some women as a place to gain information about stillbirth, although other women discussed how accessing social media in relation to pregnancy could be frightening:

*‘…so I’ve been through many reviews* [on social media], *people they say, they speak about the experience and they said, look, I’m about to give birth and the baby is dead, so it was very bad, things to read, you know… so it’s a bit scary*…Fg2.

Most women discussed difficulties migrant women may have with the spoken English language and the need for health professionals to consider this when providing key messages about reducing the risk of stillbirth:

***‘****Well if the midwife is meeting with a lady who’s not speaking English very well she should have an interpreter.’* P21, Pakistan.

The location for receiving the key messages was also highlighted with a number of women discussing how they found it helpful to receive information about pregnancy in an existing community group setting with peers and that this could be effective in communicating key messages about stillbirth prevention:

*‘there is a class I used to go to…where…pregnant women…came together and they taught us …when the baby comes how you carry them or what you have to do…like even how you sleep, how you do all that. So those classes I think really helped because…we were all involved…people from, or maybe from other countries who don’t understand English very well, so as long as someone is standing there and practising it or showing them what to do, they kind of get the idea…’* P27, Uganda.

Other group settings were discussed as a good opportunity to discuss stillbirth reduction for example ESOL classes or women’s groups in Mosques.

Women who were living in large accommodation centres for new arrivals felt that these provided an opportunity to target a group of women to discuss stillbirth prevention.

## Discussion

The risk of stillbirth is a complex issue and relates to underlying social, environmental and health factors as well as care provision and individual behaviour. The aim of this study was to consider the current awareness of public health messages relating to stillbirth prevention amongst a group of migrant women within the UK and ways in which they think these messages could be better communicated. Structural inequalities at a societal level are likely to pay a key role in the inequalities in stillbirth rates, but they are outside the remit of this study. There is evidence that widespread dissemination of consistent health messages can result in significant improvements in health outcomes, for instance the ‘*back to sleep’* campaign led to a major global reduction in sudden infant death syndrome [17]. Our findings suggest that although women were not specifically aware of receiving health messages about stillbirth reduction during pregnancy, they did recall receiving considerable advice relating to keeping their unborn baby safe from friends and family as well as health professionals. This advice was not always consistent and most participants emphasised the importance of receiving clear and trustworthy advice from health professionals.

Our findings have provided an insight into the variety of health messages that women receive about keeping their babies safe in pregnancy. We found that women have individual preferences for how stillbirth reduction messages would be best communicated including written information, use of the internet, social media and learning in group contexts. However, women believed that the health professional, in particular the midwife, is key in communicating these messages which other media can reinforce. Our study highlights the importance of the provision of a range of resources and how and when they are communicated.

Although there has been a considerable increase in focus on stillbirth prevention during the past decade, both nationally and internationally, there remains a low level of public awareness of stillbirth and the associated risk factors [18] and a continued stigmatisation of the condition [19, 20]. A lack of awareness that pregnancy complications exist is a real barrier to accepting public health messages to improve outcomes [21, 22]. Bringing stillbirth out of the shadow of silence and stigma is an important first step towards effectively communicating key messages to reduce the risk.

In order for public health messages to be accessible and acceptable, they need be communicated effectively. As our study shows, who and how information is developed and shared is important. One of the aims of our study was to understand how to ensure that key health messages to reduce stillbirth are communicated to migrant women in an accessible way. It was clear that there was no ‘one size fits all’ and that a multi-pronged approach may be the most effective, the key being consistent messages delivered in different formats and supported by trusted health professionals. This supports other research that suggests that women prefer to receive health related advice through direct interaction with health professionals [23].

A number of participants identified the need for visual as well as written materials and that many women utilise social media and online technology to access information. Mackintosh and others [2020], in their study of over 630 postnatal women in London, found that women used a range of online resources and apps to access advice and ‘self-diagnose’ potential complications in pregnancy [24]. However other research has found that current tools are not necessarily reliable, there are for instance a number of online apps for reduced movements which do not provide evidence-based advice [25]. In previous work we have found that co-produced digital animation is acceptable and accessible way of communicating evidence-based information in the perinatal period [26]. Many women in this study thought the use of short videos, viewed through their phone would be effective in backing up the key messages around stillbirth prevention communicated by the midwife.

Some women suggested that the involvement of community groups and existing support networks would support effective dissemination of key messages. This supports previous studies where utilising existing community groups to initiate discussion and share information was an effective way of communicating key public health within culturally and linguistically diverse communities, [27, 28].

Our findings highlight the importance of health professionals approaching the subject of stillbirth prevention in a sensitive manner, in particular during the antenatal period. However, as Warland and Glover [2015] argue, in order to successfully communicate these key messages, it is essential that health professionals have the resources and training to feel comfortable to talk to women from different backgrounds about stillbirth and how to keep their babies safe [29]. Focussed workshops to support maternity care providers to have these discussions have been shown to improve their knowledge and their intention to discuss stillbirth with women in their care in the future [30]. In this context, such workshops would need an emphasis on developing cultural competence and addressing potential language barriers to ensure that the key messages are acceptable and accessible. It is also worth noting that the women in this study originated from 18 different countries with heterogenous backgrounds and therefore when communicating key messages, this heterogeneity must be recognised.

## Strengths and Limitations

A key strength of this study was the engagement of women from a wide range of cultural backgrounds who had received maternity care from a range of Trusts. This helps to provide some insights into the variety and range of awareness and perceptions of stillbirth and associated public health messages. Despite this, our findings cannot be generalised to women from all communities. However, there are specific findings which can be related to women who have migrated at some point in their lives such as the influence of their home country.

Data collection was impacted by the COVID-19 pandemic and the planned focus groups were changed to individual interviews.] The plan to exploit the advantages of focus groups in using group dynamics to explore the topic in depth had to be changed [31, 32]. The initial focus groups [held with women who already knew each other outside of the research context] did not produce the level of depth that we had expected and, in reality the data that emerged from the interviews suggested that women were possibly more comfortable exploring their perceptions on an individual basis. This may have been due to the fact that they were conducted in English with informal interpreters.

Awareness of public health messages is dependent upon the messages being communicated, this can be difficult to measure as time can also impact upon recall. Nevertheless, the experience of gaps in recall may disclose a gap in the provision or delivery of the advice by health care professionals. Expanding the channels of information dissemination based upon community recommendations may positively influence health literacy.

## Conclusions

Our study highlights the complexity of discussing stillbirth during pregnancy. The women in this study recalled receiving a wide range of advice from family and friends as well as health professionals about how to keep their baby safe in pregnancy. As part of the drive to address the persistent inequalities in stillbirth rates, it is important that resources that communicate health messages to reduce stillbirth are evidence based, consistent and accessible to all communities. As our study shows, who and how information is developed and shared is relevant. Health professionals, in particular midwives who have developed a trusting relationship with the women, will be key to ensuring that public health messages relating to stillbirth reduction are communicated effectively within culturally and linguistically diverse communities.

## Supplementary information

**Additional file 1.**

## Data Availability

The dataset generated and analysed during the current study are not publicly available as they may contain information that could be confidential, but are available from the corresponding author on reasonable request.
