# Supplementary material for: An exploration of migrant women’s perceptions of public health messages to reduce stillbirth in the UK: a qualitative study
Source: BMC Pregnancy Childbirth. 2021 May 20;21:394. doi: 10.1186/s12884-021-03879-2 (PMC8136107; doi:10.1186/s12884-021-03879-2)
Supplement: Supplementary file 1 — Additional file 1. [file 12884_2021_3879_MOESM1_ESM.docx]

**Title page: Supplementary table (1)**

An exploration of Black, Asian and Minority ethnic women’s perceptions of public health messages to reduce stillbirth in the UK: a qualitative study.

**Authors**

*Tomasina Stacey

Melanie Haith-Cooper

Nisa Almas

Charlotte Kenyon

| **Key question** | **Probes** |
| --- | --- |
| Can you tell me how old you are?  Where are you from?  How long you have been in the UK?  How many children do you have?  When and where was your last birth? |  |
| Please think back to just before you were pregnant What did you **understand by the term** stillbirth? | What word would you use in your language (if English 2^nd^ language)  What does the word mean to you? |
| Please now think about when you were pregnant, what did you know about stillbirth then? | What do you remember being told about stillbirth?  Where did you get this information from? health professional, family, friend, internet, social media, religious establishment, other  How to reduce the risks of stillbirth happening  At what stage of pregnancy were you told this information?  Did you follow the advice? If not why not?  Did you get any conflicting advice? If so, what advice did you follow? |
| What suggestions do you have of the best way to provide information about reducing stillbirth? | Written or spoken? Audio visual?  Books, leaflets, internet, social media, through mobile phone?  By whom: health profession/ community member, other?  timing of advice – before/during/ after pregnancy  Location – online/ Health premises / Community space/other  What language? How could it be culturally sensitive? |
| Is there anything else that you would like to add? |  |

**Supplementary Table 1:** Interview guide
